# Pomace Olive Oil Concentrated in Triterpenic Acids Restores Vascular Function, Glucose Tolerance and Obesity Progression in Mice

**DOI:** 10.3390/nu12020323

**Published:** 2020-01-26

**Authors:** Carmen Maria Claro-Cala, Jose Carlos Quintela, Marta Pérez-Montero, Javier Miñano, María Alvarez de Sotomayor, María Dolores Herrera, Rosalía Rodríguez-Rodríguez

**Affiliations:** 1 Department of Pharmacology, Pediatric and Radiology, Faculty of Medicine, University of Sevilla, E-41009 Sevilla, Spain; jminano@us.es; 2 Natac Biotech S.L., Electrónica 7, 28923 Alcorcón, Madrid, Spain; jcquintela@natacgroup.com; 3 Basic Sciences Department, Faculty of Medicine and Health Sciences, Universitat Internacional de Catalunya, E-08195 Sant Cugat del Vallès, Spain; mperez@uic.es; 4 Departamento de Farmacología, Facultad de Farmacia, Universidad de Sevilla, E-41012 Sevilla, Spain; aldesoto@us.es (M.A.d.S.); mdherrera@us.es (M.D.H.)

**Keywords:** pomace olive oil, oleanolic acid, maslinic acid, obesity, Mediterranean diet, vascular function

## Abstract

Pomace olive oil, an olive oil sub-product, is a promising source of bioactive triterpenoids such as oleanolic acid and maslinic acid. Considering the vascular actions of pomace olive oil and the potential effects of the isolated oleanolic acid on metabolic complications of obesity, this study investigates for the first time the dietary intervention with a pomace olive oil with high concentrations of the triterpenic acids (POCTA), oleanolic and maslinic acid, during diet-induced obesity in mice. The results demonstrate that obese mice, when switched to a POCTA-diet for 10 weeks, show a substantial reduction of body weight, insulin resistance, adipose tissue inflammation, and particularly, improvement of vascular function despite high caloric intake. This study reveals the potential of a functional food based on pomace olive oil and its triterpenic fraction against obesity progression. Our data also contribute to understanding the health-promoting effects attributable to the Mediterranean diet.

## 1. Introduction

Obesity and its metabolic and cardiovascular complications represent a serious threat to the health population of almost every country in the world [1]. Not only is obesity remarkably common (13% of the adult population worldwide is obese and 39% overweight [2]) and very challenging to treat, but it is also tightly linked to insulin resistance and vascular dysfunction [3,4]. Consequently, much attention has been directed to the prevention and treatment of obesity worldwide.

Up to now, given the limited success of pharmacological interventions to effectively fight against obesity, dietary-based strategies have shown promising effects as part of the treatment of obesity and cardiovascular complications in pre-clinical and clinical studies [5,6,7,8]. In this sense, the Mediterranean diet as part of a lifestyle is considered as one of the best models of healthy eating. This traditional dietary pattern has been shown to reduce biomarkers of cardiovascular diseases and metabolic syndrome [6,9,10]. Within the Mediterranean diet, olive oil is a key component, which is partly responsible for the health-promoting effects of this diet, since it provides an excellent lipid matrix with high content in bioactive molecules of different chemical varieties [11]. In fact, identification of these molecules, the mechanisms underlying their actions and their exact contribution to the beneficial effects of olive oil and the Mediterranean diet, are of particular interest nowadays.

The cardioprotective and metabolic actions of olive oil intake have been partly attributed to their minor components [12]. Among them, the pentacyclic triterpenic acids, oleanolic and maslinic acids, have gained importance in the last years in terms of vasoprotection [13], metabolism [14,15], and cancer [16]. The procedure applied for the extraction of olive oil is crucial for the content in triterpenes and other minor constituents [17,18]. Pomace olive oil is obtained from the residue that remains after mechanical extraction of virgin olive oil. As a consequence of the extraction procedures, pomace olive oil, in spite of the lack of polyphenols, contains higher concentrations of the triterpenic fraction than virgin olive oil [18,19].

The vascular and metabolic effects of the isolated triterpenic acids have been explored both in vitro and in vivo. The endothelium-dependent vasodilatation induced by either oleanolic or maslinic acid was demonstrated in isolated arteries of normotensive and hypertensive rats [19,20]. The mechanisms mediating vasoprotection of oleanolic acid were related to activation of endothelial nitric oxide (NO) release via PI3K/AMPK/eNOS-Ser^1177^ [21] and prostacyclin release via cyclooxygenase-2 [22]. The anti-obesity potential of oleanolic acid has been found in murine adipocytes, reducing markers of differentiation and resistin production [23,24]. Besides, recent in vivo studies in mouse models of obesity revealed the beneficial effects of oral administration of oleanolic acid in glucose tolerance and visceral adiposity [25] and in body weight and fat preference in obese animals [26]. Despite these findings with the isolated oleanolic acid, the effects of the triterpenic acids on an olive oil-based diet in obesity and vascular-associated complications in vivo have not been explored.

The use of pomace olive oil with high concentrations of triterpenic acids in vivo, has been limited to our investigations on animal models of genetic hypertension [27,28,29], and the results were promising in terms of vascular endothelial function improvement [27,28], blood pressure levels attenuation, and improvement of cardiac hemodynamics [29]. Considering these vascular actions of pomace olive oil, and the potential effects of the isolated oleanolic acid on metabolic complications of obesity, our present study investigates for the first time the dietary intervention with a pomace olive oil with high concentrations of the triterpenic acids (POCTA), oleanolic and maslinic acid, during diet-induced obesity in mice. The results demonstrate that obese mice, when switched to a POCTA-diet for 10 weeks, show a substantial reduction on body weight, insulin resistance, adipose tissue inflammation, and particularly, improvement of vascular function. This study demonstrates the potential of a functional food based on pomace olive oil and its triterpenic fraction against obesity progression. Our data also contribute to understanding the health-promoting effects attributable to the Mediterranean diet.

## 2. Materials and Methods

### 2.1. Animals and Diets

Male C57BL/6 mice were obtained from the University of Seville Animal facility at 7 weeks of age. The protocol for animal handling and experimentation agreed with the European Union European Community guidelines for the ethical treatment of animals (UE Directive of 2010; 2010/63/UE) and was approved by the Ethical Committee for Animal Research of the University of Seville (RD 53/2013). Animals were fed a high-fat diet (HFD) (40% kcal from fat, Harlan, Barcelona, Spain) and water ad libitum for 11 weeks. Then, animals were randomly assigned to the following experimental groups for 10 weeks (*n* = 6–7): (1) HFD and (2) standard diet with 17% *w*/*w* of POCTA. The percentage of POCTA in the diet was calculated to reach similar caloric content from fat to that on the HFD control diet. In parallel, eleven animals were fed a standard diet (SD) for 21 weeks (*n* = 11).

The production of POCTA was according to the process of physical refining reflected in the US Patent No. US8361518 (B2), whose characteristics and composition were previously reported by Valero-Muñoz et al. [29]. The pomace olive oil used in this study has higher concentrations of triterpenic compounds than other pomace olive oils. Particularly, POCTA was concentrated in the triterpenic acids, oleanolic and maslinic acids with 4.3 and 2.87 respectively, as a percentage of total fatty acids, total sterols, and triterpenic fraction (2500 ppm) [29].

Body weight, food, and water intake were weekly evaluated. At the end of treatment, the animals were fasted for 12 h and then anesthetized and sacrificed. Blood samples were collected by an intracardiac puncture for biochemical assays in serum. A sample of visceral and epididymal white adipose tissue (vWAT and eWAT, respectively) and liver were removed and weighted, immediately frozen in liquid nitrogen, and stored at −80 °C until further analysis.

### 2.2. Blood Biochemical Assays

Serum samples were obtained from blood by centrifugation for 20 min at 4000 rpm at room temperature. Fasting glucose, total-cholesterol, and triglycerides were analyzed by UV/visible spectrophotometry kits (Spin React, CIMA Diagnostics, Girona, Spain).

### 2.3. Liver Triglycerides Quantification

Liver samples were homogenized and lipids were extracted as previously described [30]. Triglycerides were measured in the lipid extract using a commercial kit (Sigma, Madrid, Spain), following the manufacturer’s instructions.

### 2.4. Glucose Tolerance and Insulin Resistance Test

Both the oral glucose tolerance test (OGT) and the insulin tolerance test (ITT) were based on previous protocols of the group in Reference [31]. The OGT was performed by oral administration of glucose (2 g/kg body weight) to the experimental animals previously fasted for 14 h. Blood samples were obtained from the tail vein at the assay, starting in order to determine basal levels of glucose in plasma, and after 30, 90, and 120 min of glucose administration. Plasma glucose concentration was determined using a blood glucose commercial monitoring meter (Accutrend^®^Plus_GCTL; Roche Diagnostics, Barcelona, Spain). For the ITT, food was withdrawn 3 h before the test and the mice were injected intraperitoneally with insulin (100 IU/mL; Humulin Regular, Lilly S.A., Madrid, Spain). Blood samples were collected at the same time intervals. For both OGT and ITT data, each value is the total area under the glucose curve for each group of treatment.

### 2.5. RNA Extraction and Quantitative Real time-Polymerase Chain Reaction (RT-PCR) on Anti-Inflammatory Markers

Total RNA was extracted from tissues using Trizol Reagent (Fisher Scientific, Madrid, Spain). Retrotranscription and quantitative RT-PCR (qPCR) were performed as previously described [32]. The following SYBR® Green assay primers were used (IDT DNA Technologies, Leuven, Belgium): Mcp1 (for: 5′-GCTGGAGAGCTACAAGAGGATCA, rev: 5′-CTCTCTCTTGAGCTTGGTGACAAA), Tnfα (for: 5′-CCAGTGTGGGAAGCTGTCTT, rev: 5′-AAGCAAAAGAGGAGGCAACA), and β-actin (for: 5′-CGCCACCAGTTCGCCATGGA, rev: 5′-TACAGCCCGGGGAGCATCGT). Relative mRNA levels were measured using the CFX96 Real-Time System, C1000 Thermal Cycler (BioRad, Madrid, Spain). Relative gene expression was estimated using the comparative Ct (2-ΔΔct) method in relation to β-actin levels.

### 2.6. Arterial Preparation and Vascular Reactivity Experiments

The descending thoracic aorta was dissected and segments next to the aortic arch were selected and placed in modified Krebs–Henseleit bicarbonate solution (KHS), as previously described [33]. Briefly, aortic rings (1.5–2 mm in length) were mounted on a wire myograph (Danish MyoTechnology, Aarhus, Denmark) filled with KHS. Arterial segments were stretched to a resting tension of 5 mN and allowed to equilibrate for 30 min. Vasodilatation in response to the endothelium-dependent agonist, acetylcholine (ACh, 1 nmol/L–10 µmol/L), was studied in aortas with endothelium pre-contracted with the thromboxane A2 agonist (9,11-dideoxy-11α, 9α-epoxymethanoprostaglandin F2α), U46619, at 80% of their maximal response. To evaluate the involvement of NO, curves were also performed in the presence of the NO synthase (NOS) inhibitor N-nitro-L-arginine (L-NAME, 300 µmol/L). The concentration-response curves to ACh were also analyzed in the presence of the combination of L-NAME with the cyclooxygenase inhibitor, indomethacin (INDO, 10 µmol/L).

The contractile response to the adrenergic agonist, phenylephrine (Phe, 1 nmol/L to 0.1 mmol/L), was also tested in control conditions and after inhibition of the inducible NOS isoform with 1400W (5 µmol/L).

### 2.7. Data Analysis and Statistics

Results were shown as mean ± standard error of the mean (SEM). ACh-induced relaxation was expressed as a percentage of the initial contraction with U46619. Data processing and statistics were conducted using GraphPad Prism 5 Software (La Jolla, CA, USA). Statistical analysis was determined by analysis of variance (ANOVA) followed by post hoc Bonferroni test. *p* < 0.05 was considered significant. The number of animals used in each experiment is specified in each figure legend.

## 3. Results

### 3.1. POCTA Attenuated Body Weight Gain and Organ Weight in Obese Mice

**Figure 1:**
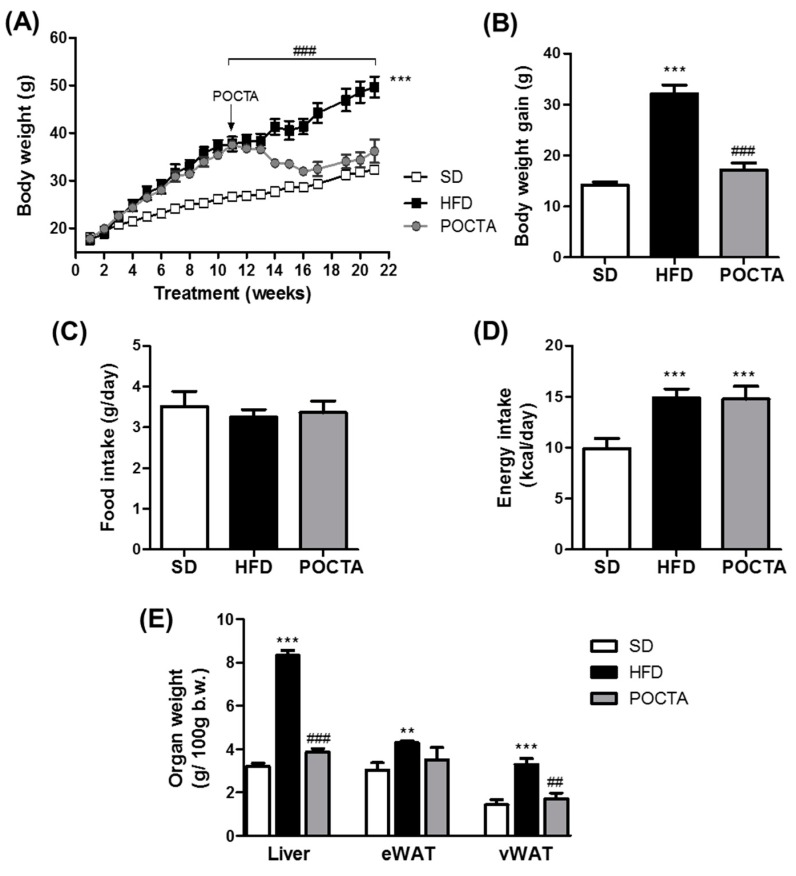
The effect of a pomace olive oil concentrated in triterpenic acids (POCTA) diet on body weight, food, and energy intake and organ weights. Body weight progression before and after the nutritional intervention in diet-induced obese (**A**), total body weight gain during 21 weeks of diet administration (**B**), food intake (**C**), caloric intake (**D**), and organ weights (**E**), of mice fed a standard diet (SD), high-fat diet (HFD), or POCTA diet. Values are mean ± SEM (*n* = 6–11) and are normalized relative to the control group. ** *p* < 0.01, *** *p* < 0.001 vs. SD; ## *p* < 0.01, ### *p* < 0.001 vs. HFD.

In order to generate the obese animal, mice were fed a HFD for 11 weeks and the results were compared to lean mice fed a SD (Figure 1).

The average initial body weight of the diet-induced obese mice on treatment week 11 was 35.92 ± 0.77 g (*n* = 13), whereas lean animals showed 26.05 ± 0.45 g (*n* = 11) body weight. At this time point, a group of obese mice were switched to a POCTA diet for 10 weeks, as illustrated in Figure 1A. This diet provided similar caloric content from fat to that provided by the HFD. The administration of the diet supplemented in POCTA during 10 weeks significantly attenuated body weight gain, reaching an average final body weight of 34.35 ± 1.47 g (*n* = 7), in contrast to the substantial increase in final body weight observed in obese mice that remained in HFD feeding (49.64 ± 2.18 g, *n* = 6) (Figure 1A,B). Body weight gain in POCTA mice was similar to that obtained in lean mice fed a SD (Figure 1A,B). Interestingly, despite the substantial attenuation of body weight gain on POCTA mice, no significant changes were appreciated on food intake compared to the rest of experimental groups (Figure 1C) and the increase in caloric intake was comparable to that observed in the HFD group (Figure 1D).

Consistent with this observation, POCTA-fed mice showed substantially lower visceral fat and liver weight than HFD-fed mice (Figure 1E). However, no statistically significant differences in epididymal fat were detected between HFD and POCTA groups (Figure 1E).

### 3.2. POCTA Improved Serum Cholesterol, Triglycerides, Glucose, and Insulin Resistance

**Figure 2:**
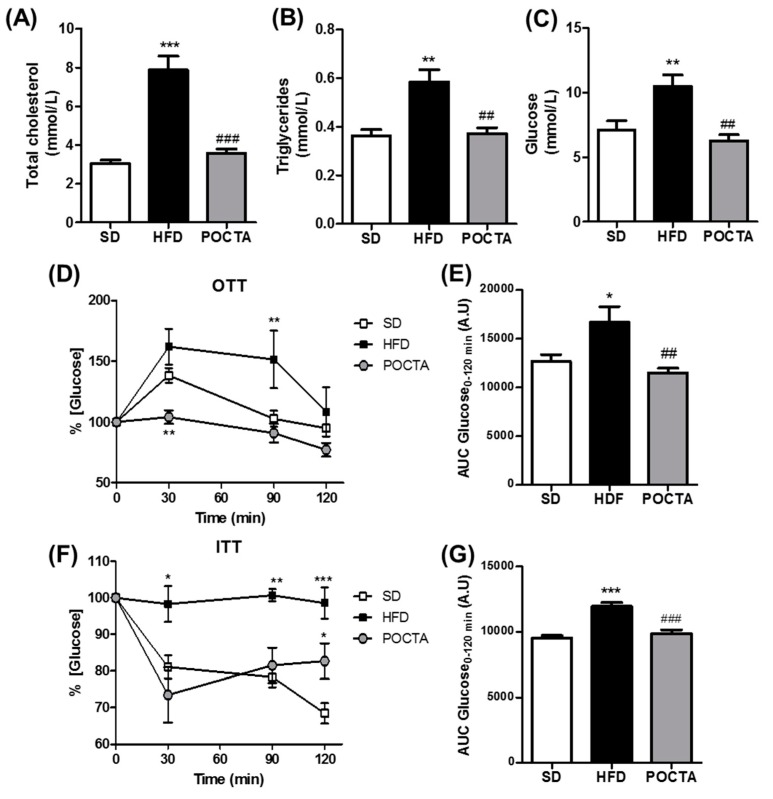
The effect of a POCTA diet on serum cholesterol, triglycerides, glucose, and insulin resistance. Levels of total cholesterol (**A**), triglycerides (**B**), and glucose (**C**) in serum of mice fed the SD, HFD, or POCTA diet. Profile of serum glucose changes obtained from oral glucose tolerance test (GTT) (**D**), and insulin tolerance test (ITT) (**F**), at 20 weeks of treatment. Area under the curve (AUC) results of serum glucose concentrations in the OTT (**E**), and the ITT (**G**). Values are mean ± SEM (*n* = 5–7) and are normalized relative to the control group. * *p* < 0.05, ** *p* < 0.01, *** *p* < 0.001 vs. SD; ## *p* < 0.01, ### *p* < 0.001 vs. HFD.

The influence of the POCTA diet on total cholesterol (TC) and triglycerides (TG) levels in serum are shown in Figure 2A,B. Levels of TC and TG in the control obese mice were significantly higher compared with those of the lean mice fed a SD. The POCTA diet decreased TC and TG levels compared to HFD-fed animals. Besides, TG levels in the liver of control obese mice were significantly higher compared with the levels of SD mice, and these levels were significantly restored by switching to a POCTA diet with the same caloric amount as the HFD (Supplementary Figure S1).

After 10 weeks of treatment, obese control mice had a high fasting blood glucose level compared to lean mice and the POCTA group (Figure 2C). Since obesity is characterized by impaired glucose tolerance and insulin resistance, the glucose and insulin tolerance tests were performed at the end of the treatment by oral administration glucose and injecting insulin intraperitoneally respectively, to evaluate the beneficial metabolic effects of the diet supplemented in the oil rich in triterpenic acids. As shown in Figure 2D, HFD-fed mice displayed hyperglycemia at 30 min, which was slightly decreased at 90 and 120 min, indicating impaired glucose tolerance, as it was confirmed by analyzing the area under the plasma glucose curve in comparison to SD-fed animals (Figure 2E). Administration of a POCTA diet to obese mice resulted in a significant improvement in glucose tolerance within 30 min of a glucose load and a reduced area under the curve (AUC) of glucose compared to the HFD group (Figure 2D).

Then, the effect of the POCTA diet on insulin sensitivity was evaluated by performing ITT. This test revealed an impaired insulin sensitivity in HFD-fed mice compared to the SD group (Figure 2F,G). Obese mice that were switched to a POCTA diet evidenced significant improvement in insulin sensitivity, as indicated by a decline in glucose levels at 30 min (Figure 2F) and a lower AUC compared to HFD mice (Figure 2G).

### 3.3. POCTA Reduced the Expression of Inflammatory Markers in White Adipose Tissues

**Figure 3:**
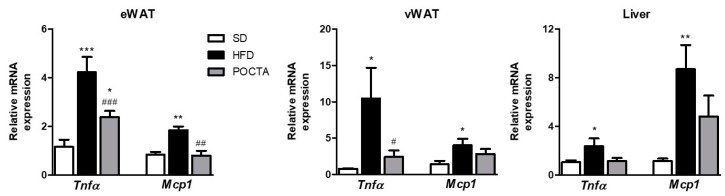
The effect of a POCTA diet on mRNA levels of pro-inflammatory cytokines (Tnf-α) and chemokines (Mcp1) in epididymal white adipose tissue (eWAT) (**A**), epididymal white adipose tissue (vWAT) (**B**), and liver (**C**), of mice fed the SD, HFD, or POCTA diet. Values are mean ± SEM (*n* = 5–7) and are normalized relative to the control group. * *p* < 0.05, ** *p* < 0.01, *** *p* < 0.001 vs. SD; # *p* < 0.05, ## *p* < 0.01, ### *p* < 0.001 vs. HFD.

In agreement with the literature, mice fed a HFD showed a significant upregulation of genes associated with inflammation in WAT and liver (Figure 3). Particularly, Tnfα and Mcp1 genes were significantly upregulated in eWAT, vWAT, and liver of HFD control mice (Figure 3). Interestingly, obese mice switched to a POCTA diet did not evidence the upregulation of these genes in either WAT depots or liver (Figure 3), and even a significant attenuation was observed on eWAT and vWAT (Figure 3A,B, respectively).

### 3.4. POCTA Restored Vascular Reactivity in Obese Mice

#### 3.4.1. Vasodilatation

**Figure 4:**
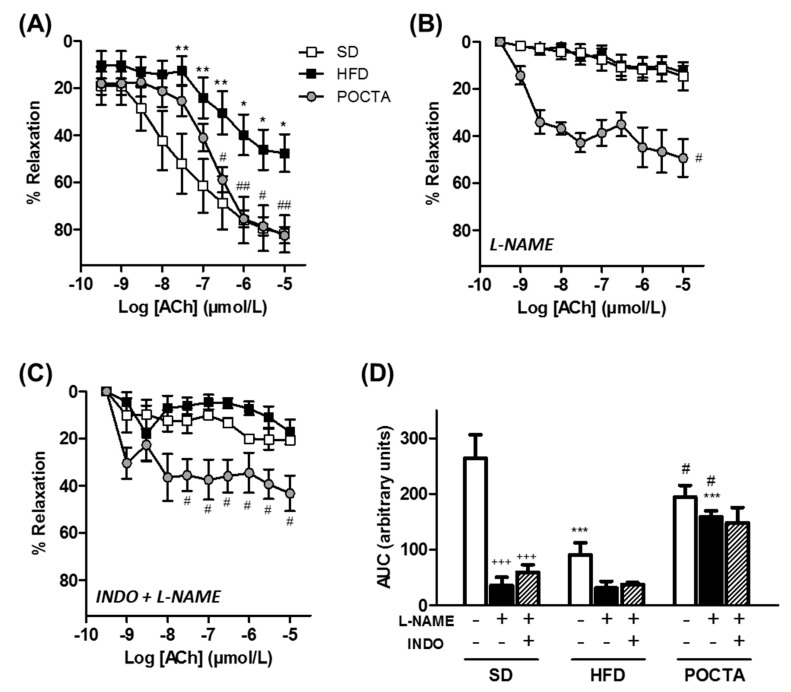
The effect of a POCTA diet on vasodilatation in aorta. Concentration-response curves to acetylcholine (ACh) in the absence of inhibitors (**A**), or in the presence of the NO synthase inhibitor N-nitro-L-arginine (L-NAME) alone (**B**), or in combination with the COX inhibitor indomethacin (INDO) (**C**), in aortic rings of mice fed the SD, HFD, or POCTA diet. Area under the curves (AUC) obtained from cumulative curves to ACh in the absence or presence of the inhibitors (**D**). Values are mean ± SEM (*n* = 5–11) and are normalized relative to the control group. * *p* < 0.05, ** *p* < 0.01, *** *p* < 0.001 vs. SD; ## *p* < 0.01, ### *p* < 0.001 vs. HFD; +++ *p* < 0.001 vs. ACh control within the same experimental group.

To evaluate the endothelial function, endothelium-dependent vasodilatation to ACh was assessed in aortic rings (Figure 4). Aortas of obese mice fed a HFD showed an impaired relaxation to ACh compared to SD mice, whereas switching to a POCTA diet significantly restored vasodilatation (Figure 4A).

To further investigate the endothelium-derived components that could be affected by either obesity or POCTA treatment in the ACh-induced vasodilatation, we examined the effect of different pharmacological inhibitors. To analyze the involvement of NO, relaxation responses to ACh were tested in the presence of the NOS inhibitor, L-NAME. Under these conditions, the ACh-evoked relaxation was abolished in both SD and HFD groups, whereas aortas of the POCTA group still showed a remaining vasodilator effect in the presence of L-NAME, suggesting a potential involvement of a NO-independent mechanism in this treatment group (Figure 4B). The contribution of the EDH-component in aortic rings was evaluated by simultaneous inhibition of cyclooxygenase (COX)-derived factors and NO synthesis by the presence of indomethacin (INDO) plus L-NAME. With both inhibitors, aortic rings of the obese mice group exhibited impairment in EDH-contribution compared to SD-fed mice, but this impairment was not that evident in the POCTA group (Figure 4C). Figure 4D illustrates the AUC of ACh dilatation curves with or without the presence of the inhibitors within the three experimental groups. Inhibition of NOS and COX pathways especially affected the vasodilatation of aortas from SD-fed mice, whereas this effect was significantly reduced in the POCTA group (Figure 4D).

#### 3.4.2. Vasoconstriction

**Figure 5:**
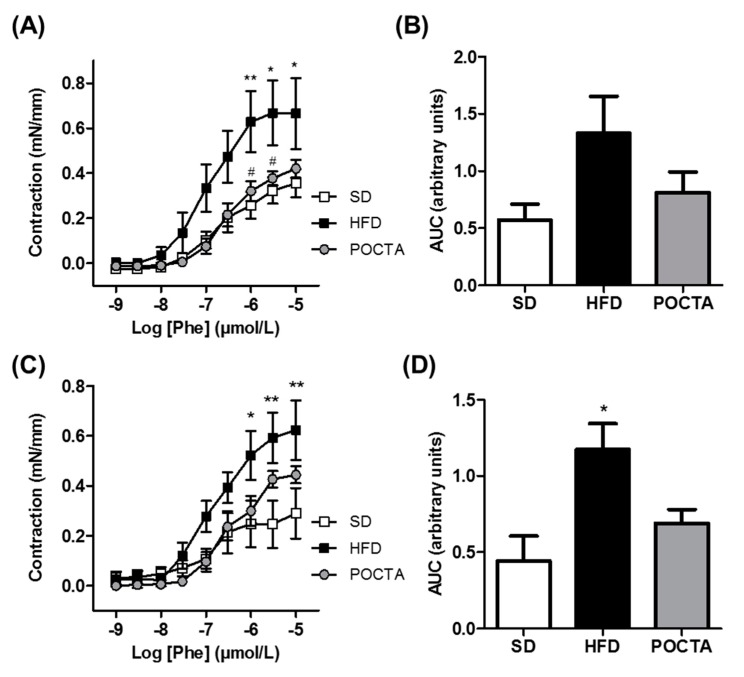
The effect of a POCTA diet on vasoconstriction in the aorta. Concentration-response curves to phenylephrine (Phe) in the absence of inhibitors (**A**, **B**), or in the presence of the inducible NO synthase inhibitor 1400W (**C**, **D**), in aortic rings of mice fed the SD, HFD, or POCTA diet. Area under the curves (AUC) obtained from cumulative curves to Phe in the absence (**B**), or presence of the inhibitor (**D**). Values are mean ± SEM (*n* = 5) and are normalized relative to the control group. * *p* < 0.05, ** *p* < 0.01 vs. SD; # *p* < 0.05 vs. HFD.

Intact aortic rings of obese control mice showed a slight increased contractile response to Phe compared to the SD group, only significantly augmented at the highest dosages of the curve (Figure 5A,B). Administration of the POCTA diet tended to attenuate this response, evidencing a similar pattern of contraction to Phe than that observed with the SD (Figure 5A,B). The differences in Phe-induced vasoconstriction between the experimental groups were maintained in the presence of the inducible NOS inhibitor, 1400W (Figure 5C,D).

## 4. Discussion

Functional foods have been proposed as a possible alternative approach of weight management and obesity prevention and of improving the cardiometabolic consequences of obesity [34]. The present investigation demonstrates the beneficial effect of a dietary-based strategy in which high fat content comes from a pomace olive oil with high concentrations of the triterpenic acids (POCTA), oleanolic and maslinic acid, in obesity- and vascular-associated complications. Previous studies from our group have shown that the consumption of POCTA restores blood pressure, endothelial function, and other risk factors related to cardiovascular diseases [27,28,29]. However, the information about the effect of pomace olive oil in alterations related to lipid and glucose metabolism and obesity remain unknown. The effects of the triterpenic acids of pomace olive oil in metabolic syndrome are limited to investigations with the administration of the isolated compounds instead of a dietary-based approach.

One of the most remarkable results in the present study is the substantial restoration of body weight gain in obese mice when switched to a POCTA diet. Interestingly, mice fed a POCTA diet showed a similar level of energy intake than that reported in the HFD-fed obese control group, indicating the relevance of the source of fat to the management of body weight. This result was endorsed by a significant attenuation of organ weights (i.e., liver and visceral WAT), serum lipids and glucose, and liver TG in mice after 10 weeks of POCTA diet administration. According to the strong association between obesity and inflammation [35], an upregulation in the expression of pro-inflammatory genes (i.e., Tnfα and Mcp1) was found in WAT and liver of obese control mice, whereas this increase was significantly attenuated in mice fed a POCTA diet. These data suggest a potential effect of POCTA on WAT and liver-specific prevention of obesity. In line with these evidences, the isolated triterpene oleanolic acid has shown anti-obesity effects either in vitro or in vivo, since it regulates adipogenesis, lipolysis, and fatty acid oxidation in preadipocytes [36], downregulates the expression of adipogenic factors, and ameliorates visceral adiposity in diet-induced obese mice [25,37]. Maslinic acid has also been recently shown to modulate glucose uptake and lipid metabolism in adipocytes [38] and hepatic cell lines [15]. In vivo, intraperitoneal administration of maslinic acid for 12 weeks significantly reduced body weight and non-alcoholic fatty liver disease in diet-induced obese mice [15].

The effect of the high caloric POCTA diet on body weight management and adiposity in mice could be explained by the positive results of this diet administration on the lipid and glucose profile, and particularly on insulin resistance. Obese mice fed a POCTA diet showed a significant improvement in oral glucose tolerance and intraperitoneal insulin sensitivity compared to obese control mice. It is important to mention that recent evidences reported that triterpenic acids such as oleanolic acid act as hypoglycemic agents mainly by (i) reducing the absorption of glucose, (ii) decreasing endogenous glucose production and increasing glycogen synthesis, (iii) increasing insulin sensitivity, and (iv) improving lipid homeostasis [39]. In addition, oleanolic acid and its biotransformed metabolites are potential α-glucosidase inhibitors [40]. Recent studies with animal models of obesity reported that chronic administration of oleanolic acid modulates fat preference and inflammation [26], ameliorates visceral adiposity, and improves blood glucose tolerance in mice fed a HFD [25]. The fact that the administration of a POCTA diet to obese mice attenuated pro-inflammatory genes in adipose tissue and liver, which are strong markers of insulin resistance and systemic inflammation, also supports the role of POCTA in obesity-associated glucose tolerance and insulin resistance.

Obesity implies the development of vascular abnormalities, in particular impaired vasodilatation in various vascular beds, that affects vascular homeostasis and the delivery of substrates to metabolically active tissues [41]. Vascular dysfunction related to obesity seems to be derived from several changes in adipose tissue, leading to a chronic inflammatory state and dysregulation of adipocyte-derived factors and consequently, an imbalance between the vasoprotective (e.g., NO) and the vascular hazardous factors (e.g., endothelin-1) [41,42]. In addition, hyperinsulinemia and insulin resistance contribute to vascular abnormalities since the balanced endothelium-dependent vasodilator and vasoconstrictor effects of insulin are shifted towards predominant vasoconstriction in obesity [43,44]. In the present investigation, a significant impairment in endothelium-dependent vasodilatation was observed in HFD-fed obese mice. This endothelial dysfunction associated with obesity was clearly restored by administration of a POCTA diet, with potential involvement of either NO- or EDH-dependent mechanisms. In line with this finding, our group previously reported that long-term administration of a diet supplemented in POCTA improved ACh-mediated vasodilatation in either aorta or resistance arteries of hypertensive rats [27,28]. The mechanisms underlying these actions involved NO- and EDH-dependent pathways [21,27,28].

In addition to impaired vasodilatation, obesity is associated to an increased vascular tone in response to different stimuli [45,46]. This increased vascular contraction in obesity has been related to an elevated endothelin-dependent signaling and an augmented adrenergic stimulation in blood vessels [45,46]. Administration of a POCTA diet slightly attenuated Phe-induced vasoconstriction, reaching a similar level of constriction to that found in SD-fed mice. The use of a selective inhibitor of the inducible NO synthase indicated a contribution of this isoform on aortic contraction in obese mice, whereas the POCTA diet appeared to attenuate inducible NO synthase involvement. The vasoprotective effects found after administration of a POCTA diet in obese mice are in line with the significant improvement in serum lipid and glucose profile, fat inflammation, and insulin resistance.

In summary, this study demonstrates that obese mice, when switched to a diet in which the main source of fat is POCTA for 10 weeks of administration, showed a significant attenuation in obesity progression and associated complications. These effects of POCTA could be mainly attributable to the high content on triterpenic acids, which have separately demonstrated anti-obesity effects and vasoprotection in animal models of obesity and cardiovascular diseases. Remarkably, POCTA is particularly interesting since pomace olive oil is a major sub-product of olive oil production that results from an eco-friendly system and its composition is a lack of polyphenolic fraction but high in bioactive triterpenoids. Although the mechanisms underlying the beneficial effects of a POCTA diet on metabolic syndrome are probably multiple, in our study, we demonstrated for the first time that POCTA can restore body weight gain, adipose tissue and liver inflammation, insulin resistance, and vascular dysfunction associated with obesity. In addition, it is important to mention that body weight management in mice fed a POCTA diet was observed even though the animals showed the same level of food and caloric intake compared to the HFD control diet. The results of the present investigation provide insight into the therapeutic potential of the traditional use of pomace olive oil as a source of bioactive triterpenic acids. Since pomace olive oil has been considered as a waste product to the olive oil industry, and this study contributes to increasing the biological and nutritional value of POCTA as a functional food against metabolic syndrome. Taking into account the lack of clinical studies with pomace olive oil in obesity and metabolic syndrome, it would be of great interest to carry out clinical studies to extend the understanding on the health benefits derived from sustained pomace olive oil intake [47].

## Supplementary Materials

The following are available online at https://www.mdpi.com/2072-6643/12/2/323/s1, Figure S1: The effect of a POCTA diet on liver triglycerides in mice fed a standard diet (SD), high-fat diet (HFD), or a diet supplemented in olive pomace oil with high concentration in triterpenic acids (POCTA).
