# Supplementary material for: Pomace Olive Oil Concentrated in Triterpenic Acids Restores Vascular Function, Glucose Tolerance and Obesity Progression in Mice
Source: Nutrients. 2020 Jan 26;12(2):323. doi: 10.3390/nu12020323 (PMC7071211; doi:10.3390/nu12020323)
Supplement: Supplementary file 1 [file nutrients-12-00323-s001.pdf]

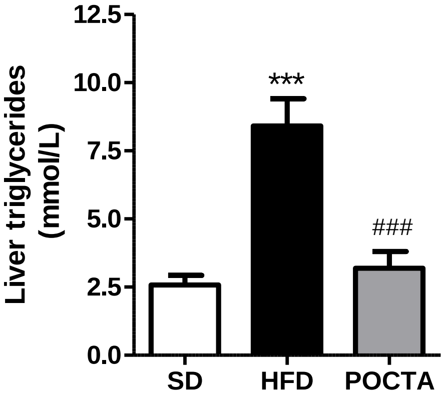


**Figure S1.** The effect of a POCTA diet on liver triglycerides in mice fed a standard diet (SD), high fat diet (HFD) or a diet supplemented in olive pomace oil with high concentration in triterpenic acids (POCTA). Values are mean ± SEM (n =5-10) and are normalized relative to the control group. ***$P<0.001$ vs SD; ###$P<0.001$ vs HFD.
